# A Tale of Two Proteolytic Machines: Matrix Metalloproteinases and the Ubiquitin–Proteasome System in Pulmonary Fibrosis

**DOI:** 10.3390/ijms21113878

**Published:** 2020-05-29

**Authors:** Willy Roque, Alexandra Boni, Jose Martinez-Manzano, Freddy Romero

**Affiliations:** 1 Department of Medicine, Rutgers—New Jersey Medical School, 185 S Orange Ave, Newark, NJ 07103, USA; wr160@njms.rutgers.edu (W.R.); ajb311@njms.rutgers.edu (A.B.); 2 Brigham and Women’s Hospital—Pulmonary and Critical Care Medicine, Boston, MA 02115, USA; martinezmanzano.jm@gmail.com; 3 Department of Medicine, Division of Pulmonary, Allergy and Critical Care and the Center for Translational Medicine, The Jane & Leonard Korman Respiratory Institute, Philadelphia, PA 19107, USA

**Keywords:** pulmonary fibrosis, ubiquitin-proteasome system, metalloproteinases, proteostasis, lung aging

## Abstract

Pulmonary fibrosis is a chronic and progressive lung disease characterized by the activation of fibroblasts and the irreversible deposition of connective tissue matrices that leads to altered pulmonary architecture and physiology. Multiple factors have been implicated in the pathogenesis of lung fibrosis, including genetic and environmental factors that cause abnormal activation of alveolar epithelial cells, leading to the development of complex profibrotic cascade activation and extracellular matrix (ECM) deposition. One class of proteinases that is thought to be important in the regulation of the ECM are the matrix metalloproteinases (MMPs). MMPs can be up- and down- regulated in idiopathic pulmonary fibrosis (IPF) lungs and their role depends upon their location and function. Furthermore, alterations in the ubiquitin-proteosome system (UPS), a major intracellular protein degradation complex, have been described in aging and IPF lungs. UPS alterations could potentially lead to the abnormal accumulation and deposition of ECM. A better understanding of the specific roles MMPs and UPS play in the pathophysiology of pulmonary fibrosis could potentially drive to the development of novel biomarkers that can be as diagnostic and therapeutic targets. In this review, we describe how MMPs and UPS alter ECM composition in IPF lungs and mouse models of pulmonary fibrosis, thereby influencing the alveolar epithelial and mesenchymal cell behavior. Finally, we discuss recent findings that associate MMPs and UPS interplay with the development of pulmonary fibrosis.

## 1. Introduction

Pulmonary fibrosis refers to a group of conditions that cause scarring of the lung. It is estimated that these conditions represent 10% of all visits to subspecialty pulmonary clinics, making fibrotic lung conditions one of the major causes of morbidity and mortality from respiratory illness. Although outcomes vary considerably depending on the etiology of fibrotic lung disorder, these conditions can be quite devastating, causing respiratory failure and death in a significant number of patients [1]. Currently, there are a limited number of only marginally effective treatment options for patients with progressive forms of fibrotic lung disease, emphasizing the need for further mechanistic insight and translational progress [2]. There are over 200 different types of pulmonary fibrosis, and in most cases, there is no known cause. Some cases of pulmonary fibrosis are caused by environmental exposures, such as asbestos or silica, and others occur in individuals with genetic predispositions [3]. While IPF is considered a unique form of pulmonary fibrosis, it has long been recognized that a nearly identical condition develops in middle-aged individuals called the Hermanksy-Pudlak Syndrome (HPS). Unlike idiopathic pulmonary fibrosis (IPF), the etiology of HPS is known, as this condition results from autosomal recessive mutations in one of 10 lysosomal trafficking proteins. Despite these differences, the pathologic changes in pulmonary fibrosis in HPS is nearly indistinguishable from IPF, exhibiting similar lung fibroblast activation and exaggerated accumulation of extracellular matrix (ECM) components such as collagens and fibrins [4]. These pathologic changes are likely due to protease dysregulation, specifically matrix metalloproteinases and the ubiquitin proteasome system, leading to the protein aggregation evidenced in these diseases.

Matrix metalloproteinases (MMPs) are an integral component of multidirectional communication between the cells and the ECM. These proteinases have a broad spectrum of substrates, and their regulation is, therefore, essential to maintain the integrity of the lung architecture. Upregulation of MMPs has been described in IPF and HPS lungs and is considered a direct contributor to the abnormal remodeling seen in the lung microenvironment, not only for their dysfunctional breakdown of products but also by activating growth factors and cytokines that lead to migration, host defense, proliferation, angiogenesis, and apoptosis [5]. The production and activity of MMPs are regulated at the level of transcription and by various post-translational mechanisms, with activation of the zymogen and inactivation of the active proteinase being among the most critical.

Additionally, several studies have shown that inhibition of UPS suppresses the activity of several MMPs [6,7,8,9,10]. The ubiquitin-proteasome system (UPS) is responsible for the degradation and clearance of 80% to 90% of all misfolded, oxidized, and damaged intracellular proteins [11,12]. It uses a highly regulated stepwise process through ubiquitin-ligases enzymes to tag proteins destined to be cleared by the proteasome. The UPS serves as a significant quality control regulator of multiple intracellular processes that modulate the ECM composition, including expression of specific MMPs and collagen by transcriptional and posttranscriptional modifications [13]. As a result, modulation of the UPS activity is being considered a potential pharmacological approach to fine-tune MMPs expression and reduce tissue remodeling in pulmonary fibrosis.

This review explores the main aspects of MMPs and UPS regulation, the evidence establishing an inter-play between these two, their role in the pathogenesis of pulmonary fibrosis and potential therapeutic targets for this devastating disease.

## 2. Matrix Metalloproteinases

The MMPs are zinc-dependent endopeptidases that carry a diverse range of functions, including being the activation-regulators of numerous proteins. MMPs provide direct cleavage of diverse mediators such as growth factors, cytokines, chemokines, and antifibrotic growth factors. In mice, 23 MMPs genes have been described, while humans express 24 MMPs genes, including two genes for MMP23, though the mammalian orthologue of MMP18 has not yet been identified. Variations in the structure are seen according to the type of MMP and specific function, which can include a hemopexin domain, transmembrane domain, or glycophosphatidyl-inositol (GPI) anchor, fibronectin-binding site or furin-cleavage site [14]. MMPs are therefore best classified based on structure. MMP activity has a strong, multi-level regulation process, consisting of transcriptional/post-translational changes and localization within the cell. Transcriptional regulation of MMP gene expression follows multiple elements: growth factors, cytokines, hormones, cell adhesion molecules, ECM proteins, and mRNA stability. Researchers have also classified MMPs into three categories based on the nature of promoter binding sites: MMPs with a TATA box and AP binding sites, MMPs with a TATA box but not AP binding sites, and MMPs lacking a TATA box. MMPs with different promotor binding sites vary by tissue location. Post-translational modifications are crucial, MMPs are produced initially as proenzymes, which are not functional until they pass through an activation mechanism called a cysteine switch [15].

The activation of pro-MMPs occurs upon direct proteolytic cleavage of the pro-domain (within the secretory pathway or extracellularly) or induction of conformational changes that disrupt the chelating cysteine residue and enable auto-proteolysis for removal of the pro-domain region. Allosteric activation of pro-MMPs can also be triggered by interaction with ECM components, interaction with cell surface molecules, and low-density lipoprotein receptor-related protein (LRP1)-mediated endocytosis [16,17,18]. Upon activation, mature MMPs are controlled by endogenous inhibitors, such as tissue inhibitors of metalloproteases (TIMPs) and α2-macroglobulin [19].

TIMPs are multifunctional proteins that regulate MMPs activity extracellularly in the tissue microenvironment. Four homologous TIMPs have been described: TIMP-1, -2, -3, and -4. These proteins offer inhibitory functions by binding noncovalently to MMPs and are also associated with cellular proliferation, cell survival, anti-angiogenesis, and apoptosis induction [19,20]. In pulmonary fibrosis, high levels of TIMPs were reported in immunohistochemical studies, identifying the presence of TIMP-1 in interstitial macrophages, TIMP-2 in fibroblast foci within the alveolar walls, TIMP-3 in the elastic lamina of vessels, while TIMP-4 was expressed mainly in alveolar epithelial cells, interstitial macrophages, and plasma cells [21,22,23]. While more and new studies are required to elucidate the clear role of TIMPs, these findings highlight the potential role of these proteins in the modulation of the ECM remodeling seen in lung fibrosis. Furthermore, compartmentalization of MMPs within the cell to either the cytosol or bound to the surface allows regulation by preventing accidental activation and protects active MMPs from inhibition by tissue inhibitors of MMPs (TIMPs). A clear example of this type of regulation is the role of MMP9, which has two forms: secreted and surface-bound, and localization of this protein allows it to carry distinct functions [24]. Moreover, the role of the MMPs in shaping ECM remodeling is complex. It has been hypothesized that abnormal fibrotic remodeling is a consequence of improper homeostasis between the deposition and degradation of ECM elements. Moreover, recent studies suggest that overexpression of certain MMPs in IPF can influence the activation pro-fibrotic and anti-fibrotic mediators given that individual MMPs made by different cell types carry out specific functions. For instance, specific MMPs made by AEC could be pro-fibrotic, whereas the same MMP made by myofibroblast could be anti-fibrotic. Thus, these proteins play a more broadened role in the pathogenesis of this disease [25,26].

## 3. MMPs in Pulmonary Fibrosis

The “mechanistic convergence model” postulates that IPF is caused by the simultaneous occurrence of multiple processes that lead to a distinctive pathogenic flow, resulting in the inappropriate stimulation of alveolar epithelial cells [27]. MMPs play a crucial role in the aberrant fibrotic tissue remodeling, but the precise mechanisms are yet to be fully characterized. Upregulation of several MMPs in human and experimental models of lung fibrosis have been reported in prior studies [28], as shown in Table 1. Relevant to this, recent work has shown that MMP1 is overexpressed in IPF lungs and has been associated to the pathogenesis of this disease. MMP1 can degrade fibrillar collagens, which are heavily accumulated in molecules that make up the ECM in IPF. This seems counterintuitive, as an antifibrotic mediator is upregulated lung parenchyma, yet the lungs in IPF, by definition, become fibrotic. This can be explained by the preferential expression of MMP1 in different lung tissue: MMP1 overexpression is localized to the alveolar epithelial cells (AEC) and lung macrophages, allowing fibrosis elsewhere in the interstitial lung compartment [29,30,31,32]. Furthermore, MMP3 is believed to be involved in the pathogenesis of IPF by inducing epithelial to mesenchymal transition. This theory is based on studies showing that MMP3 is upregulated in both IPF lungs and bleomycin-challenged mice and that MMP-3 knockout mice do not develop lung fibrosis after bleomycin challenge [5,33]. MMP3 leads to fibrosis in the lungs by activating several pathways, including the cleavage of E-cadherins via β catenin signaling, TGF-β release, and releasing endostatin bound to type XVIII collagen. While initially considered to be essential to promote re-growth of the ciliated epithelium of the airway, it is now appreciated that sustained activation of MMP7 underlies the pathobiology of pulmonary fibrosis. This is based on studies in which mice lacking MMP7 were protected from lung fibrosis induced by bleomycin [34]. Similar changes in MMP7 levels have been observed in the lung epithelium of patients with IPF, suggesting that MMP7-targeted therapies may have a future in the treatment of human fibrotic disease. Consistent with an essential role in the regulation of ECM components, a recent study showed that MMP7 cleaves osteopontin, an important cytokine that helps to modulate cell adhesion and migration [35].

Another important MMP that has been shown to be upregulated in IPF lungs is MMP9, specifically in AEC, macrophages, neutrophils, and fibroblasts [21,48,49,50]. Despite several reports of MMP9 in IPF, whether this MMP contributes to lung remodeling or takes part in the reparative response, remains to be determined. Under normal conditions, MMP9 is present in low levels in the healthy adult lung; this is thought to be due to thy-1, a glycoprotein that is expressed in normal lungs but not in IPF lungs. Thy-1-deficient fibroblasts produced MMP9 when exposed to TGF-β1, and treatment of these cells with a TGF-β1 antagonist (β glucan) prevented MMP9 production. MMP9, in turn, activates TGF-β into its functioning form. Thus, MMP9 and TGF-β1 are likely regulators in pro-fibrotic feedback loops contributing to IPF pathogenesis. Additionally, it has been shown that MMP19 is highly expressed in epithelial cells surrounding fibrotic tissue in IPF lungs. Notably, these studies also uncovered the mechanism by which MMP19 regulates the production of ECM and fibroblast transmigration, namely through augmenting the activation of prostaglandin endoperoxide E2 and the levels of anti-fibrotic prostaglandin E2 [51]. Further, mice lacking MMP19 had a more robust fibrotic response than wild type mice. In sum, these enzymes carry pleiotropic effects, their role in the pathogenesis of IPF goes beyond remodeling the ECM, their influence in cytokines and growth factor regulation needs still to be comprehensively studied to identify novel and safe therapeutic targets.

**Figure 1:**
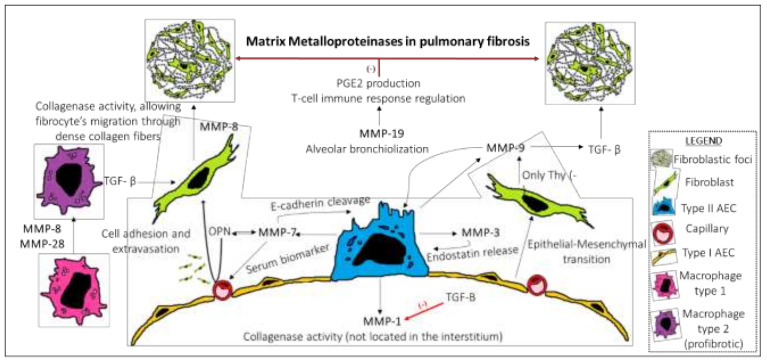
Proposed model of MMPs role in pulmonary fibrosis. Alveolar epithelial type 2 (AE2) cells play a central role in the pro-fibrotic response after repeated lung epithelial injury through several mechanisms, including the upregulation of several matrix metalloproteinases (MMPs). MMP-1 has collagenase activity; however, the location of this protease is not in the interstitium and does not break down the extra-cellular matrix (ECM) proteins. Additionally, MMP-1 is downregulated by TGF-β. MMP-3 has a role in the transformation of epithelial cells to mesenchymal cells, which are producers of ECM proteins, such as type I collagen. MMP-3 is also involved in the release of endostatin, which induces AEC apoptosis. MMP-7 has a crosstalk interaction with osteopontin (OPN), which enhances the MMP-7 production and promotes endothelial adhesion and extravasation of cells, including fibrocytes, which are a precursor of tissue fibroblasts. MMP-7 cleaves E-cadherin and contributes to AEC activation. Additionally, MMP-7 has been validated as a serum biomarker in IPF. MMP-8 has collagenase activity and allows fibrocytes to migrate through interstitial collagen fibers. MMP-9 is produced by AE2 and Thy (-) fibroblasts and enhances the activity of TGF-β, which is a growth factor related to the stimulation of fibroblasts and interstitial fibrosis development. MMP-9 is also related to abnormal alveolar bronchiolization. MMP-19 has antifibrotic properties linked to the production of prostaglandin E2 through the stimulation of prostaglandin-endoperoxide synthase 2. Regulation of adaptative immune cell response has also been related to MMP-19 antifibrotic effects. MMP-28 promotes the macrophage M1-M2 phenotypic switch, as well as MMP-8.

Dysregulated MMPs activity has been described in the lungs of HPS. We previously reported that mutations in two different HPS genes (HPS1 and HPS2) lead to upregulation and increased activity of MMPs. Our study showed that HPS2-mice had elevated transcript levels of MMP2 and MMP9, while broad upregulation of MMP2, -3, -8, -9, -12, and -14 transcripts was evidenced in the HPS1-mice, suggesting that the pathogenesis of pulmonary fibrosis might differ in the different HPS mutations. Interestingly, we found that elevated MMPs levels in HPS lung are related to chronic activation of serine/threonine kinase, Akt. Activation of this enzyme has been associated with fibrotic tissue growth, proliferation, and metabolic programming of fibroblasts. In addition, Akt activity has been shown to be upregulated in alveolar epithelial type 2 (AE2) cells of IPF lungs, supporting the notion that targeting Akt might represent a therapeutic approach in HPS and IPF [52] (Figure 1).

## 4. The Ubiquitin-Proteasome System

In mammalian cells, the primary mechanism by which intracellular proteins are degraded is through the UPS. This system provides the degradation of 80% to 90% of proteins within the cell. Clearance of misfolded, oxidized, and damaged proteins is accomplished through the actions of the UPS, making it crucial to maintain cellular proteostasis [53]. Degradation of proteins by the UPS requires a stepwise process. First, target proteins go through ubiquitination by receiving a covalent linkage of ubiquitin to a lysine 48 (K48) residue. Ubiquitination is an ATP-dependent process that requires the action of three different enzymes: E1, a ubiquitin-activating enzyme that forms a thioester bond with the ubiquitin protein, allowing it to bind to another enzyme, the E2 ubiquitin-conjugating enzyme. This leads to the formation of an isopeptide bond between the carboxy-terminus of ubiquitin and lysine residue on the target protein. Ultimately, the E3-ubiquitin ligase provides high specificity for individual substrates for the ubiquitin-binding onto the target protein. Second, once proteins are ubiquitinated, they are delivered to the 26S proteasome, a large, 2.5-MDa, located mainly in the cytoplasm, designed to perform proteolysis. The 26S proteasome contains two main structures: the 20S particle (core particle, CP) and a regulatory 19S complex (regulatory particle, RP). The 20S is formed of a barrel proteolytic core with ATP-dependent activity; it consists of duplicating seven α subunits and seven β subunits that form four axially stacked heteroheptameric rings. The outer-α rings have seven subunits (alpha1-alpha7) that act as a bridge regulating the entrance and removal of degraded proteins, while the inner-β rings have seven subunits (beta1-beta7) where protein breakdown takes place, along with the release of amino acids and peptide fragments into the cytoplasm for recycling. To date, the three types of protein lysis have been described in the 20S proteasome: 1) chymotrypsin-like (CT-L); 2) trypsin-like (T-L), and 3) caspase-like (C-L). The 19S is divided into the base and the lid. The base serves as a regulatory element with six regulatory AAA ATPase subunits (Rpt1-Rpt6) and four non-ATP subunits; instead, the lid consists of nine Rpn subunits and functions to remove ubiquitin molecules. The specific regulatory mechanism of proteasomal gene expression has not been fully elucidated [54,55,56].

Current evidence suggests that either the expression of these proteins might be controlled by one main transcription factor or require several other regulatory proteins. Most recently, studies have identified two essential transcription factors in many proteasome genes: Rpn4 and the nuclear factor erythroid 2-related factor 1 (Nrf1) [57]. Interestingly, one recent study reported that the mechanistic target of rapamycin (mTORC1) could increase proteasome biogenesis, suggesting that free amino-acid supply secondary to proteasomal protein destruction plays a vital role during the activation of mTOR-mediated pro-growth pathways [34,58]. In sum, proteasome activity is a complex and tightly regulated process influenced by multiple factors; the understanding of these mechanisms is critical to elucidate the elements that modulate the cellular proteome.

## 5. UPS in Pulmonary Fibrosis

A hallmark of age-related diseases is the failure in proteostasis, leading to the accumulation of protein aggregates [59,60]. The decline of proteasome activity has been observed in different aged cells with subsequent accumulation of misfolded, aggregated, and oxidized proteins. The mechanism that leads to proteasome dysfunction in aging is associated with decreased expression or alteration of proteasome subunits, disassembly of proteasomes, and inactivation of the proteasome by protein aggregates [61,62,63]. As a consequence of these proteome alterations, a series of pathways can be activated in pursuit of re-establishing proteostasis: the heat-shock protein response (HSR), integrated-stress response (ISR), the unfolded protein response (UPR) or the mitochondrial unfolded protein response (UPRmt). In age-related diseases, a decline in the HSR has been reported, contributing even more to the accumulation of unfolded proteins that oversaturate the proteasome machinery [64]. Interestingly, expression of the chaperone HSP70 is regulated by the HSR activation; this protein interacts with STUB1 (previously known as CHIP), an E3-ubiquitin ligase to promote unfolded protein degradation [65,66]. A study by Min et al. reported that the deletion of STUB1 in mice leads to accelerated aging phenotypes [67]. Moreover, Xin et al. found that STUB1 decreases SMAD3 expression through the actions of the UPS [68]. While the precise mechanism is still unclear, it suggests that this E3-ubiquitin ligase might inhibit TGF-B signaling. Certainly, these studies provide a meaningful connection between heat-shock proteins, UPS activity, aging, and fibrosis.

Alveolar epithelial cells and fibroblast of IPF patients are characterized by severely dysregulated proteome and consequently the altered concentrations of multiple proteins. Persistent, dysregulated, and widespread epithelial injury sustains the production of collagen and other ECM components that create the chronic and ineffective wound healing response. In the pathophysiology of IPF, secretion of TGFβ1 has been identified as a significant driving factor in the progression of the fibrotic process by promoting differentiation of fibroblast into myofibroblast, resulting in abnormal ECM deposition [69,70,71]. Several downstream mediators of this factor are regulated through the UPS [72,73]. For instance, levels of K48-ubiquitination were increased in human lung fibroblasts following TGFβ1 exposure; the same study demonstrated that levels of the proteasome subunit Rpn6 were increased in fibroblast of IPF patients [74]. In addition, it was demonstrated that mice treated with a proteasome inhibitor, Bortezomib, were protected from the damaging effect of bleomycin [75]. Increased proteasome activity was also evidenced in total lung extracts of IPF patients when compared to healthy donors [11]. These findings suggest that proteasome activity modulation can become a therapeutic target in lung fibrosis by inhibiting fibroblast activation through TGFβ1 signaling. However, further studies in AEC are necessary to fully understand whether proteasome activity is increased or decreased in this cell line, in addition to elucidating how these changes influence proteome alterations in age-related lung fibrosis.

Recent evidence suggests the UPS is a primordial factor in the protein quality control of distinct organelles, specifically the mitochondria [76]. Mitochondrial dysfunction is considered a pathogenic trigger in the development of age-related diseases, including IPF [77]. UPS activity seems to regulate mitochondria biogenesis by the degradation of mTORC1, which increases the expression of PGC-1a. The secretion of mTORC1 leads indirectly to the translation of mitochondrial proteins, including complex V, complex I, and Tfam. It is possible that once mTORC1 action is completed, this protein is tagged by an E3-ubiqituin ligase and delivered to the proteasome for degradation [78]. Mitochondria dynamics are also regulated by the actions of the UPS. Cherok et al. described the role of MARCH5 and SUMO-1, both E3-ligases that recruit Drp1 to influence mitochondria fission [79]. Similarly, other E3-ligases have been implicated in the regulation of mitochondrial fusion; these include the ubiquitin ligase Skp1-144 Cullin-F-box (SCF-Mdm30) and MULAN/MAPL by tagging Mfn1 or Mfn2 for degradation [80]. These suggest that part of the alterations in mitochondria dynamics and dysmorphic mitochondria accumulation found in mouse models of IPF could be attributed to alterations of the UPS. As previously mentioned, mitochondrial dysfunction is considered a major pathogenic factor in age-related diseases, including IPF. The accumulation of dysmorphic/dysfunctional mitochondria in AE2 is associated to impaired mitophagy secondary decreased activity of PTEN-induced putative kinase 1 (PINK1). Interestingly, in mice models of bleomycin-induced pulmonary fibrosis, increased susceptibility to lung fibrosis was found secondary to PINK1 deletion, in addition to dysfunctional mitochondria and protein aggregation in the endoplasmic reticulum (ER), suggesting a link between mitochondria function and proteostasis alteration in the pathogenesis of this disease [77,81].

## 6. The Interplay between the UPS and MMPs: Therapeutic Perspective

As stated earlier, 80% to 90% of proteins in eukaryotic cells are degraded by the UPS actions [53]. Additionally, this system regulates multiple molecules involved in the turnover of ECM, including the MMPs via transcriptional and posttranslational modifications, establishing a bridge between intracellular to extracellular protein degradation. The interplay between MMPs and the UPS has been explored in several organs [82,83,84,85]. Modulation of this system may be considered a novel therapeutic approach to reduce tissue remodeling in IPF. For instance, Ortiz-Lazareno et al. reported that proteasomal inhibition using MG-132 leads to reduced secretion of pro-fibrotic and pro-inflammatory cytokines (TNF-*α*, IL-1*β*, IL-6) by U937-macrophages [86]. Similarly, a study by Mao et al. used a murine model of inflammation by giving Titanium (TI) particles and subsequent administration of Bortazemib; as a result, decreased expression of TNF-*α*, IL-1*β*, IL-6 was also evidenced [6]. These findings highlight the proteasome as a critical mediator in perpetuating the fibrotic process by regulating the secretion of these growth factors/cytokines that allow fibroblast/myofibroblast proliferation, angiogenesis stimulation, and ECM production. Moreover, inhibition of proteasomal activity using MG132 in rat myocardial fibroblasts prevented the expression of profibrotic MMP-2, MMP-9, and collagen, which limited cardiac remodeling and fibrosis of cardiac tissue [85]. Further, degradation of type collagen-1 is accomplished by MMP-1, and evidence suggests that TGFβ1 secretion downregulates MMP-1 while upregulating type I collagen, a protein identified as a major component in lung scan formation. In human fibroblasts, inhibition of the proteasome leads to increase in MMP-1 production while reducing type I collagen formation [7]. Interestingly, we recently reported that individual HPS mutation leads to differences in proteasome activity of lung fibroblasts, where HSP2-mice had increased levels of proteasome proteins and activity, while the opposite is seen in HSP1-mice. This implies that there is a need for individualized therapeutic strategies for patients with specific HPS subtypes when targeting the proteasome. In this context, ECM remodeling and degradation can be modulated by the actions of the proteasome, suggesting that modifications in the UPS activity could effectively reduce tissue remodeling in lung fibrosis [52,87].

**Figure 2:**
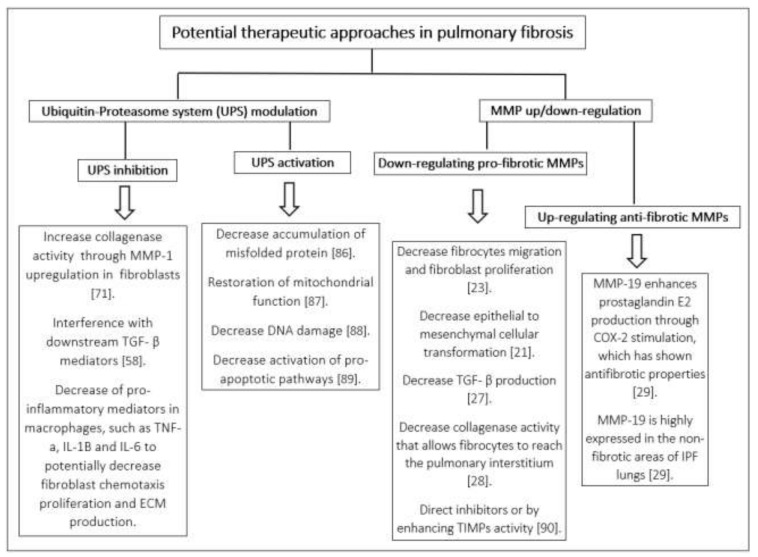
Potential therapeutic approaches in pulmonary fibrosis involving the ubiquitin-proteasome system (UPS) and metalloproteinases (MMPs) modulation. COX: cyclooxygenase, IL: interleukin, TGF-β: tumor growth factor β, TIMPs: tissue inhibitors of metalloproteinases, TNF: tumor necrosis factor, IPF: idiopathic pulmonary fibrosis.

On the other hand, targeting MMPs as a therapeutic strategy has also been explored. Corbel et al. reported that in a mice model of bleomycin-induced lung fibrosis treatment with Batismastat, a synthetic MMP inhibitor, the levels of hydroxyproline, MMP-2, MMP-9 and histopathological findings of fibrosis were reduced [8]. Moreover, an immunization-based strategy to target MMPs was reported in two different studies. Sela-Passwell et al. used a molecular mimicry approach by producing inhibitory antibodies showing TIMP-like mechanism leading specific blockage of MMP-9 and MMP-14 activity [9]. Similarly, Marshal et al. developed a selective allosteric MMP-9 inhibitor, the humanized monoclonal antibody GS-5745, proposing an innovative strategy to target MMPs [10]. Further, selective upregulation of MMP-13 and MMP-19 by enhancing the expression of their transcription factors was proposed as a target due to their antifibrotic properties. Nonetheless, no significant experimental studies have been performed to test this hypothesis [88]. Further studies are still needed to delineate the specific protein alterations in IPF lungs, their regulation, and the mechanism by which each one contributes explicitly to the disease pathogenesis. Nonetheless, together these findings support the notion that therapeutic strategies designed to regulate the activity of certain MMPs by modulating the proteasome activity could be beneficial in the treatment of IPF (Figure 2).

We hypothesize that targeting Nrf1/Tcf11, the major regulator of proteasome subunits expression, might constitute a promising therapeutic approach in pulmonary fibrosis by interplaying between the UPS, MMPs, and the Wnt/B-catenin pathway. During lung development, activation of the Wnt/B-catenin plays a key role in cellular fate, cellular migration, angiogenesis, and apoptosis [89,90]. Aberrant re-activation of the Wnt/B-catenin pathway in alveolar epithelial cells contributes to the pathogenesis of IPF through numerous mechanisms, including the upregulation of MMP-2 and MMP-9 [91,92,93,94]. Moreover, several E3-ligases target B-catenin for ubiquitin-proteasome mediated degradation, including β-TrCP, Mule, Jade-1, Siah-1, Hakai, and Ozz-E3 [90,95,96]. Interestingly, a recent study by Chen et al. showed that overexpression of Nrf1 enhanced B-catenin ubiquitination by the E3 ligase B-TrCP and led to subsequent proteasomal degradation. Conversely, the authors reported that knockdown of Nrf1 leads to increased mRNA expression of MMP-2 and MMP-9 [97]. These results demonstrate that enhancing Nrf1 expression could indirectly inhibit the pro-fibrotic response of MMP-2 and MMP-9, making this transcription factor an attractive therapeutic target.

## 7. Concluding Remarks

Over the years, MMPs have proven to be crucial determinants in the pathogenesis of lung fibrosis. Evidence suggests that the role of the MMPs goes beyond remodeling the ECM components. Further, overexpression of certain MMPs in IPF leads to the activation of pro-fibrotic and anti-fibrotic mediators, hence contributing to the improper homeostasis seen between deposition and degradation of ECM elements. Despite significant gaps in present knowledge about MMPs, these are still a promising target to restore the integrity of the lung architecture. Moreover, considering the complex biological alterations involved in the pathogenesis of pulmonary fibrosis, including misfolded protein aggregation, it is reasonable that restoring protein homeostasis within the cell by targeting the UPS, the major protein degradation system, is a viable approach. We hypothesized that modulating the activity of the UPS could arrest disease progression and re-establish part of the lung architecture. Nonetheless, further studies are required to understand the specific molecular mechanism that regulates MMPs expression and UPS activity before developing safe and effective therapies. Further directions for research could also include the roles of MMPs and UPS dysregulation in patients with IPF and concomitant respiratory infections such as tuberculosis, lung cancer, or chronic obstructive pulmonary disease, as both MMPs and the UPS have been implicated in those diseases.

## Figures and Tables

**Table 1:** Upregulated Metalloproteinases in Pulmonary Fibrosis. AEC: alveolar epithelial cells, COX: cyclooxygenase, EMT: epithelial-mesenchymal transition, TGF-β: tumor growth factor β, IPF: idiopathic pulmonary fibrosis.

	Pro/Anti-Fibrotic Action	Studies in Transgenic Animal Models	Cellular Source	Mechanism of Action	IPF Biomarker	Outcome
**MMP-3**	Pro	Yes. Bleomycin	AEC, fibroblasts, alveolar macrophages	EMT [33] Wnt/B-catenin pathway activation [33] Endostatin release	No	Aberrant cellular repair. Fibrocytes extravasation. CD103+ dendritic cells.Myofibroblast proliferation. Re-epithelization by alpha(2) beta(1) integrin activation [36].
**MMP-7**	Pro	Yes. Bleomycin	AEC	Osteopontin production [37] E-cadherin cleavage [38] Syndecan-1 shedding [39]	Yes	
**MMP-9**	Pro	Yes. Bleomycin	AEC, fibroblasts	Abnormal alveolar broncholization. [40] Induced by TGF-B in Thy (-) fibroblasts [41]	No	
**MMP-28**	Pro	Yes. Bleomycin	Macrophages	Switch M1-M2 phenotype and TGF-B production [42]	No	Fibroblast stimulation by M2 macrophages.
**MMP-8**	Pro	Yes. Bleomycin	Fibrocytes, macrophages, AEC and several other immune cells	Collagenase activity [43]	No	Fibrocyte migration through dense collagen fibers and posterior maturation to fibroblast
**MMP-19**	Anti	Yes. Bleomycin	AEC in spared areas in IPF lung areas.	COX-2 stimulation and PGE32 production exert antifibrotic effects [44] Regulation of adaptive immune response [45]	No	Spared lung tissue around fibrotic zones.
**MMP-1**	Anti	No	AEC	Collagenase Inhibits apoptosis [30] Promotes normal re-epithelization of wounds	No	Inhibited by TGF-B and osteopontin in IPF lungs [37].
**MMP-13**	Pro and Anti	Yes. Bleomycin and Radiation	Fibroblasts	Increased lung inflammation with Bleomycin [46] Decreased inflammation with radiation [47]	No	Not clear role in IPF lungs.
